# On the Use of the Speculum

**Published:** 1851-02

**Authors:** 


*On the Use of the Speculum.* By Prof. C. R. Gilman.

The *New York Journal of Medicine and the Collateral Sciences*, No. for Jan., 1851, contains an able article by Prof. Gilman on the use of the Speculum. The distinguished author prefaces the consideration of the subject by remarks upon the motives which prompt, on the part of some, opposition to improvements in the early stage of their introduction—while they may be stigmatised as innovations. Who does not recollect the ridicule and disrepute which so simple and innocent an instrument as the stethoscope had to encounter! It is a satisfaction, however, to know that the *true* and the *useful* must, in the end, triumph; and that, generally, clamor and opposition by awakening attention, and exciting investigation, often tend to promote the interests of science.

We transfer to our columns the portion of the article by Prof. Gilman which relates directly to the use of the Speculum.

EDITOR BUFFALO MEDICAL JOURNAL.

The speculum, we hoped, might now be allowed to take its place beside the stethoscope, a valuable means of investigating and treating disease—a means by which one of the *opprobria medicorum* was removed, and leucorrhea made a curable disease. But it seems it is not to be so. The attack upon this instrument made in London, finds a ready echo in our own land, and we are called to fight this battle over again, and to oppose here, as in the case of chloroform, facts and experience to reasonings, theories, and declamation. The contest is not one from which the advocates of the speculum need shrink. The result is not doubtful.

The opponents of the speculum, aware that it would hardly be safe to deny outright its value, have taken other, and as they doubtless suppose, more tenable ground: they profess not to forbid, but only to limit its use; "it is of great value in proper cases, but it has been used unnecessarily and for bad motives, and we must limit its use." To this limitation some of those who know the speculum practically will accede, especially as regards malignant disease. But they rely on its use in simple inflammation and ulceration; here the speculum is as useful in the treatment as in the diagnosis of disease. How is its use to be *limited* in this class of affections? Simply by denying the alleged frequency, and some go as far as to doubt the *existence*, of simple ulceration. This is the ground taken in London by Drs. Lee and Ashwell. The proofs relied on to support this most extraordinary assertion are—1st, That in very many *post mortem* examinations of women dying of other diseases, no ulcerations were found by the various curators of museums by whom these examinations were made. The number of such examinations rises to thousands, and might no doubt be carried to tens of thousands; for, as Dr. Bennett well observes, when these examinations were made, the practical knowledge of the inflammatory lesions of the uterus did not exist in the profession. But, 2d, The opponents of the speculum refer to their own experience to prove the extreme unfrequency of simple ulceration. Dr. R. Lee has not seen a single case of simple ulceration of the cervix. Dr. Ashwell, in 1026 cases of uterine disease treated by him at Guy's, found only 25 cases of ulceration of the cervix.

This reference to facts and cases is just what the friends of progress desire: it is ground they are accustomed to tread, and over which they move with assured steps. Let it then stand recorded, that of 1026 cases of uterine disease treated at Guy's Hospital, London, only 25 are found with ulcers of the cervix. This is a very valuable fact. Let us collect other facts, and compare the experience of other men with that of Dr. Ashwell. Dr. Murphy, in the same debate, said that he had seen hundreds of uterine cases, and seven-tenths of them were inflammations and ulcerations of the cervix. Dr. Bennett, in 300 cases, found diseased cervix in 243, ulceration in 222. Here, indeed, is a marvellous discrepancy!

Dr. Murphy finds diseased cervix in seven-tenths of his cases, Dr. Bennett in five-sixths, Dr. Ashwell in one-fortieth, and Dr. R. Lee never a single case! How is it possible to account for such discrepancies? Can any plausible explanation be offered other than that suggested by Dr. B.?—*They did not use the speculum.*

Is not this the obvious, the inevitable conclusion from the premises? The simple truth is—it must be—these gentlemen have not seen ulceration, because they have not looked. They do not know, because they will not learn. Now what could the advocates of the speculum desire more than this: in Guy's Hospital the number of cases of simple ulceration that are not diagnosticated, and of course not well treated, is seven-tenths less one-fortieth, or about sixty-five per cent. Here we have, in a tangible shape, the fruits of this doctrine of the abuse of the speculum. To avoid such abuse of the speculum, Dr. Ashwell will not use it, and he fails accurately to diagnosticate sixty-five per cent. of his cases. Here is an abuse indeed. But will any one charge me with disrespect to Dr. Ashwell? Let the treatment of Bennett, and other less distinguished men who think with him, be my defence, if I need one. Did not Dr. R. Lee assert that he did not believe Dr. B. had ever seen a simple ulcer of the cervix; and that after Drs. Locock and Murphy had declared that they met with them very frequently? And is not this the talk of all those who rail at the abuse of the speculum? Dr. Bennett, Dr. Locock, Dr. Murphy, the hundreds, might I not say thousands of practitioners who, in Great Britain, on the continent of Europe, and in this country, a part of whose daily business it is to see, and treat, *and cure* these ulcerations, are laboring under some strange hallucination, for to this we are driven—there is no room for mistake. I see a case of chronic leucorrhea; the patient is incapable of exertion, her health is broken, her spirits depressed to the lowest point, for she has been for years under treatment—has tried tonics without number, and washes without end, and all to no effect; I use the speculum. I *think* I see the cervix large, red, and on either lip I *imagine* that I see an ulcer. Under the influence of this idea—supposing, nay, so far has the delusion gone with me, verily believing that there is an ulcer—I apply nitrate of silver, I cauterize this *supposed* ulcer. I do this again and again; my patient gradually improves in health—is convalescent—is well. She returns to her home, a happy, useful wife; and in a year or two I get a letter, full of gratitude and happiness—the long barren wife is a mother. Now is this case—and, like many others who devote special attention to diseases of the uterus, *and use the speculum*, my experience will supply many such—is this case a mere delusion? Have I dreamed that I saw this ulcer—that, after repeated cauterizations, I saw it growing less and less, till nothing but sound mucous membrane appeared? Have I dreamed all this? Must I, at the dictum of Dr. Lee, or Dr. Ashwell, or Dr. any-body-else, give up my own observation, my own experience? And why? Because they will not look, and do not see. But there are great moral considerations, and Dr. Ashwell says he should feel tempted to give up the treatment of the diseases of women altogether, if the speculum continued to be used as it has been. And shall I follow his example?—shall I give up the instrument that has enabled me to restore scores of women to health and happiness, because it is indelicate? Well may Dr. Locock say that the talk about the indelicacy of the use of the speculum was all nonsense. Nonsense it is—poor, paltry, but yet mischievous, most mischievous nonsense; and, for myself, I believe that all this talk about treating the use of the speculum is little better, and most of that about its abuse is very much worse. The attempt to cast reproaches on honorable men for well-intended efforts to advance our science and improve our means of curing disease, because those attempts involve personal exposure, is much more likely to have its origin in professional jealousy than in moral principle.

P. S. Since this was written, I have seen Marshall Hall's letter. He says, " a woman on whom the speculum has been used is never the same, *morally*, she was before." Did this come from another man, I should feel it my duty to the many excellent women for whose benefit I have used it, to say, that a grosser calumny on female purity never was uttered.

